# Do Interpersonal Conflict, Aggression and Bullying at the Workplace Overlap? A Latent Class Modeling Approach

**DOI:** 10.3389/fpsyg.2018.01743

**Published:** 2018-10-09

**Authors:** Guy Notelaers, Beatrice Van der Heijden, Hannes Guenter, Morten Birkeland Nielsen, Ståle Valvetne Einarsen

**Affiliations:** ^1^Faculty of Psychology, University of Bergen, Bergen, Norway; ^2^Institute for Management Research, Radboud University, Nijmegen, Netherlands; ^3^Department of Organisation, Open University of the Netherlands, Heerlen, Netherlands; ^4^Kingston Business School, Kingston University, London, United Kingdom; ^5^Maastricht University School of Business and Economics, Maastricht University, Maastricht, Netherlands; ^6^National Institute of Occupational Health, Oslo, Norway

**Keywords:** bullying, aggression, conflict, latent class factor modeling, latent class modeling

## Abstract

In this paper, we tackle an important but unresolved research question: How distinct are workplace conflict, aggression and bullying? We study this question by means of latent class (LC) analysis using cross-industry data from 6,175 Belgian workers. We find a two-factor solution (conflict-aggression versus bullying) to provide the best fit to the data. Employees with low exposure to conflict-aggression and bullying perceived the phenomena as mostly overlapping. Employees who were exposed more frequently to the phenomena reported them to be more distinct - especially so for workplace bullying. We also find conflict-aggression and bullying to have distinct relationships with well-being and strain outcomes. These findings entail that a simple unifying approach or a single label for all three phenomena is not appropriate, at least from a measurement point of view and from the perspective of those exposed. Our results have important implications for the theoretical understanding of conflict, aggression and bullying, and for practitioners who provide support to affected employees including policymakers who help prevent and manage these problems at the workplace.

## Introduction

Notwithstanding the enjoyment people experience from their job and the effort and engagement they put into it, work can also have negative effects. Social stressors at work like interpersonal conflict, aggression and workplace bullying have been found to have severe negative consequences for employees, organizations, and society, for example, by negatively affecting employee commitment, engagement, and health (Bowling and Beehr, 2006; Nielsen and Einarsen, 2012). Prior research has studied these social stressors in depth but has typically focused on one of those stressors at work only, with few studies trying to understand their commonalities and differences (Hershcovis, 2011; Hershcovis and Reich, 2013). Despite a lack of primary studies investigating conflict, aggression, and bullying in conjunction, meta-analytical evidence has provided support for the potential overlapping nature of these phenomena and some scholars have called for a unifying approach (Hershcovis, 2011; Hershcovis and Reich, 2013). To better understand in how far conflict, aggression, and bullying overlap, we examine whether employees experience these issues at the workplace as distinct or as overlapping phenomena, and whether they relate differently to individual health and well-being outcomes.

This study has important theoretical, methodological, and practical implications. With regard to theory, the study sheds light on the basic question of whether conflict, aggression, and bullying are labels that can be used interchangeably referring to the same underlying phenomena. With regard to methods, it introduces the Latent Class (LC) analysis as a framework for the empirical identification of both the overlap and the distinctness of constructs. As regards practice, the study provides insights for managers and HR practitioners as well as for law and policy-makers on how to address, label and handle those social stressors at work.

### The Very Nature of the ‘Beasts’

Workplace conflicts, aggression and bullying refer to work-related interpersonal problems, all of which have some features in common but also differ in many regards, as obvious from their definitions. Interpersonal conflict describes a “process that begins when one party perceives that the other has negatively affected, or is about to negatively affect, something that he or she cares about” (Thomas, 1992, p. 653). Workplace aggression is commonly referred to as “any behavior directed toward another individual that is carried out with the *proximate* (immediate) intent to cause harm” (Anderson and Bushman, 2002, p. 28). That is to say, to qualify as workplace aggression, the perpetrator must expect the behavior to cause harm in the target and must believe that the target is motivated to avoid this behavior. Bullying at work, most commonly, is defined as “harassing, offending, socially excluding someone or negatively affecting someone’s work tasks. In order for the label bullying (or mobbing) to be applied to a particular activity, interaction or process, it has to occur repeatedly and regularly (e.g., weekly) and over an extended period of time (e.g., 6 months). Bullying is an escalating process in the course of which the person confronted ends up in an inferior position and becomes the target of systematic negative social acts” (Einarsen et al., 2011, p. 15). Across definitions, bullying is consistently described by the exposure to frequent and systematic negative and unwanted behaviors at work, mainly exhibited by colleagues or superiors, and mainly with acts of a psychological nature (Olweus, 1978, 1991; Leymann, 1996). Behaviors involved are often of subtle and indirect nature, usually resulting in the social exclusion of the target (Björkqvist et al., 1994a; Einarsen et al., 2011).

As evident from these definitions, it seems that there is considerable overlap among interpersonal conflict, aggression and bullying. This seems not that surprising given that they all describe interpersonal behaviors at work that can cause harm to others. Consequently, Bowling and Beehr (2006), in a meta-analysis, grouped studies on interpersonal conflict, workplace aggression and bullying together under the umbrella concept of workplace harassment. In a similar Schat et al. (2006) grouped similar constructs together under the umbrella of workplace aggression. Although conflict researchers, up until now, have not argued for such a unifying approach, they tend to conceive bullying as yet another conflict (De Dreu et al., 2001), that merely signals an extreme case of conflict (De Dreu et al., 2004). In line with the latter, Van de Vliert (2010) reformulated workplace bullying as a conflict that is blown up, broader and longer-lasting than other conflicts (p. 87).

Conflict, aggression, and bullying, while sharing some commonalities, also differ in some important ways (Spector and Fox, 2005; Aquino and Thau, 2009) (see **Table 1** for an overview). Aggression and bullying differ from interpersonal conflicts in that they entail uniformly *negative behaviors* that the target is highly motivated to avoid. To the best of our knowledge, no positive outcomes have been recorded for aggression and bullying (Nielsen and Einarsen, 2012), at least not for those targeted. Interpersonal conflicts, in difference, may also entail positive behaviors and outcomes (Raver and Barling, 2008), which, however, may show only under specific but rather rare conditions (De Dreu, 2008). For instance, moderate task-related conflicts can have positive *outcomes* as they improve group-decision making and prevent groupthink (Janis, 1972; Nemeth, 1986; Cosier and Schwenk, 1990; Amason, 1996; Brodbeck et al., 2002). Such positive outcomes, however, are exclusive to task conflicts and not found for relationship conflicts (Guenter et al., 2016).

**Table 1:** Similarities and differences across constructs (reworked from Raver and Barling, 2008; Hershcovis, 2011).

	*Nature of behavior*	Duration of behavior	Power differences	Intent to harm	Outcomes
Conflict	Psychological Positive Negative	Episodic or persistent	No	No	Negative and positive
Aggression	Psychological/Physical Negative	Episodic	No	Yes	Negative
Bullying	Psychological Negative	Persistent (long term)	Yes	No	Negative


Aggression differs from interpersonal conflicts and bullying in that aggression often manifests in physical ways such as shoving, hitting and, kicking (Buss and Perry, 1992; Felson and Tedeschi, 1993; Einarsen et al., 2009). Conflicts and bullying, in difference, mostly manifest in verbal and non-physical ways (such as by not including someone or not communicating in situations where that may otherwise be the case or appropriate to do so). Conflicts and bullying are marked by behaviors, which, if seen in isolation, may not appear to be highly adverse but which unfold their truly negative effects if repeated, which is a defining characteristic of bullying, but not of conflict (Einarsen et al., 2011, p. 15; Baillien et al., 2017).

Aggression also differs from conflict and bullying in that it is enacted with the *intent to harm* others (Neuman and Baron, 2003; Schat et al., 2006; Raver and Barling, 2008). When comparing definitions of concepts related to workplace bullying (Keashly and Jagatic, 2003; Raver and Barling, 2008; Hershcovis, 2011; Notelaers, 2011), diversity is found which is attributable to the different approaches used among European and American scholars (Keashly and Jagatic, 2003). No single definition by European researchers explicitly denotes ‘intent’ as a defining element in workplace bullying. This is nevertheless the case in some definitions by American scholars. Here it should be noted that although the particular behaviors may not be intended to cause harm, this does not mean that bullying is not harmful. Obviously, either actual intent and/or perceptions of intent may be involved. Both longitudinal and meta-analytical studies find bullying to cause severe harm to individuals and organizations (Høgh et al., 2011; Nielsen and Einarsen, 2012; Nielsen et al., 2014). Similarly, a conflict (that has gone “wrong”) may be harmful as well. Thus, aggressive behavior is harmful by its intention (harm is established *a priori*), while conflicts and bullying are harmful because of their potential consequences (harm is established *a posteriori*).

Bullying differs from interpersonal conflict and aggression in that it is characterized by a pattern of repeated behaviors over an extended period of time, whereas conflict and aggression can manifest in a single interaction (Olweus, 1993; Leymann, 1996; Einarsen, 2000; Keashly and Jagatic, 2003; Notelaers et al., 2006a). Hence, the *duration of the behavior* is different. Bullying is the result of a process where a power imbalance between the target and the perpetrator exists or emerges; due to this power imbalance the target is unable to defend him or herself against the treatment (Olweus, 1993, 2003; Zapf, 1999; Einarsen, 2000; Zapf and Gross, 2001; Van de Vliert, 2010). As such, time plays an important role for all three constructs, albeit to a varying degree. That is, conflicts may escalate or de-escalate over time (Glasl, 1994; van de Vliert, 1998; De Dreu et al., 2004) and targets of aggression may report multiple aggressive episodes (Keashly and Jagatic, 2003). In order to qualify as bullying, the negative encounters need to stretch over a lengthy period.

Although conceptually, conflict, aggression, and bullying seem to have many noteworthy similarities (and differences), there is a shortage of empirical studies on their convergence (and divergence) (Aquino and Thau, 2009). To our knowledge, only three previous studies have examined conflict, aggression, and bullying in conjunction. However, these studies have not investigated the extent to which these constructs are related to each other. Using a multi-method design in a cross-sectional study, Ayoko et al. (2003) found the three concepts to be related although they focused mainly on investigating aggression and not on exposure to aggression. In a Spanish sample, Leon-Perez et al. (2013) identified clusters to separate exposure to bullying from exposure to aggression, but they did investigate interpersonal conflicts as a criterion variable only. Finally, Hershcovis (2011) presented meta-analytical support for the notion that conflict, aggression, and bullying are strongly related. Based on the correlations between conflict, aggression and bullying with measures of job satisfaction, physical and psychological well-being, affective commitment and turnover intention, the author concluded that the constructs were overlapping in the majority of cases.

### Research Hypotheses and Analytical Strategy

From a definitional point of view, conflict, aggression and bullying have many communalities. According to Hershcovis (2011) the field has reached a point at which construct proliferation and overlap demand a synthesis. Indeed, a key issue identified within a professional development workshop at the 2008 Academy of Management conference (Raver, 2008) was the concern that the field is becoming fragmented. While numerous overlapping constructs were developed, these largely examine the same relationships.

Hershcovis argued that there is more that unites conflict, aggression, and bullying than differentiates them. Hershcovis and Barling (2007) and Raver and Barling (2008) raise similar concerns. As indicated here above, Schat et al. (2006) grouped similar constructs together under the umbrella of workplace aggression when providing prevalence estimates for the USA. Hence it seems reasonably to assume that conflicts, aggression and bullying resort under one conceptual umbrella.

Hypothesis 1: Conflicts, aggression and workplace bullying items are indicators of one single overarching concept (Model 1).

Using LC analysis, we estimate one single factor or a n-latent class cluster model wherein latent classes of respondents may be identified according to their nature and exposure to all the indicators (Notelaers et al., 2006a, 2011; Einarsen et al., 2009). To test Hypothesis 1, we will compare the fit of this model with the fit of alternative measurement models.

In line with the suggestion that aggression is a form of conflict (Raver and Barling, 2008; Keashly and Jagatic, 2011), we first estimate the fit of a two-factor model that distinguishes between one factor comprising both conflicts (C) and aggression (A), and another factor reflecting workplace bullying (WB) (Model 2a – CA-WB). Following the assertion that aggression and bullying are different labels for a common concept (Hershcovis, 2011; Hershcovis and Reich, 2013), we also estimate a two-factor model that distinguishes conflict from aggression-bullying (Model 2b – AWB-C). Following the line of reasoning that bullying is a(n) (escalated) form of conflict (De Dreu et al., 2004; Van de Vliert, 2010), we also estimate a model wherein conflicts and bullying are conceived of as one unified factor, with aggression constituting a separate factor (Model 2c – A-CWB). Finally, in line with Tepper and Henle (2011) who argue that it is the differences between constructs that define them, we estimate a three-factor model that differentiates between conflicts, aggression, and workplace bullying in order to test whether these concepts actually reflect three different and distinct phenomena (Model 3 – A-C-WB).

Previous research has found rather similar correlations between conflict, aggression, and bullying on the one hand, and job satisfaction, turnover intention, psychological well-being, physical well-being and affective commitment on the other hand (Bowling and Beehr, 2006; Hershcovis, 2011). We thus expect these three types of social stressors at work to be equally detrimental.

Hypothesis 2: Conflict, aggression and bullying have similar negative effects.

To test hypothesis 2, we investigate the unique predictive validity of conflict, aggression, and bullying on well-being and strain outcomes. As prior scholarly work has associated conflict, aggression, and bullying with reduced commitment and job satisfaction, sleeping problems, stress, and turnover (Keenan and Newton, 1985; Spector and Jex, 1998; Frone, 2000; De Dreu et al., 2002; LeBlanc and Kelloway, 2002; Bowling and Beehr, 2006; Nielsen and Einarsen, 2012), we will use similar criterion variables.

## Materials and Methods

This study is based upon a re-analysis of data on workplace bullying published earlier (Notelaers et al., 2006b). However, by including both interpersonal conflicts and aggression in addition to bullying, and hereby studying the occurrence of, and the interrelationships between, all three phenomena simultaneously, we extend the previous study and present novel findings of great importance to the field.

### Procedure and Sample

The sample is a convenience sample, which consists of 6,175 employees from 19 different, and highly heterogeneous working organizations. The 19 organizations that participated had sought contact with the Belgian Health and Safety Executives (HSEs) who, by Belgian law, are entitled to guide organizations and employers with respect to their prevention policies regarding safety, ergonomics, health, and well-being. The Directorate Research of Working Conditions of the Belgian Federal Department of Labour treated the data and made reports for the HSEs in order to ensure their further commitment to future studies on psychosocial factors. Participation of the specific individual employees was voluntary and anonymity was guaranteed by all parties involved. None of the members of the participating organizations had access to any of the completed surveys, herewith fully guaranteeing anonymity.

In the final sample, 44.5% of the respondents were female. Their mean age was 40.5 years (*SD* = 10) and their mean tenure was 12 years (*SD* = 11). Approximately 7.7% of the respondents were blue collar workers, whereas 31.8% were white collar workers. About 8% of the employees were nurses or assistant nurses, and one out of three respondents were public servants. Almost 8% of the respondents occupied a lower managerial role versus 11% being in a higher managerial role. The respondents were distributed as follows over different branches: 22% manufacturing industry, 37% services, 33% governmental services, and 7% public health.

### Measures

To measure perceived *conflicts* with colleagues and leaders, two items from the Questionnaire on Experience and Evaluation of Work were employed (van Veldhoven and Meijman, 1994): (1) ‘Do you have any conflict with your colleagues?’ and ‘Do you have any conflict with your direct boss/supervisor?’ Four answering categories were offered to the respondents: ‘never,’ ‘sometimes,’ ‘often,’ and ‘always.’

To measure *aggression* from colleagues and leaders, two items from the Questionnaire on Experience and Evaluation of Work (van Veldhoven and Meijman, 1994) were employed: (1) ‘Do you experience any aggressiveness on the part of colleagues?’ and ‘Do you experience any aggressiveness from your direct boss?’ Four response categories were used: ‘never,’ ‘sometimes,’ ‘often,’ and ‘always.’

To measure exposure to workplace *bullying*, six items were selected from the Short Negative Acts Questionnaire. The SNAQ measure is a quasi-unidimensional measure with three main types of indicators, that is, work-related acts, person-related acts, and acts of social isolation. The selected items had the highest factor loadings in a confirmatory LC factor analysis (Notelaers and Einarsen, 2008). Example items are: ‘Repeated reminders about your mistakes’ (work-related act), ‘Repeated reminders about your blunders’ (person-oriented act) or ‘Social exclusion from co-workers or work group activities’ (act of social isolation). Respondents were asked to indicate how often they had been subjected to these behaviors during the last 6 months. The response categories were: ‘never,’ ‘now and then,’ ‘once a month’ and ‘once a week or more often.’ As the items are ordinal indicators of a LC model consisting of nominal or ordinal latent variables, we did not calculate Cronbach’s alpha.

*Symptoms of well-being and work strain* were also measured with the aforementioned Questionnaire on Experience and Evaluation of Work. A total of 38 items of this questionnaire were used, measuring six indictors of symptoms of well-being and work strain. Response alternatives were: ‘yes’ or ‘no.’ *Job satisfaction* was measured with nine items like: ‘I dread going to work’ (reversed coded) and ‘I’m pleased to start my day’s work.’ Internal stability as measured by Cronbach’s alpha (α) was 0.84. *Organizational commitment* was measured using eight items (α = 0.75) like: ‘I really feel very closely involved with this organization’ or ‘I feel very at home working for this organization.’ *Turnover intention* was measured with four items (α = 0.76). Example items are: ‘I sometimes think about seeking work outside this organization’ or ‘Next year, I plan to change jobs.’ *Need for recovery* was measured using eleven items (α = 0.88) (e.g., ‘I find it difficult to relax at the end of a working day’ and ‘Because of my job, at the end of the working day I feel absolutely exhausted’). *Worrying* was measured using four items (α = 0.81): ‘When I leave my work, I continue to worry about work problems,’ and ‘I often lie awake at night pondering about things at work.’ *Sleep quality* was measured using fourteen items (α = 0.90) such as: ‘I often do not get a wink of sleep at night’ and ‘At night, more often than not, I am tossing and turning.’

### Statistical Considerations

Previous research on interpersonal conflicts, aggression, and bullying has mainly relied on standard linear regression techniques. This kind of approach has some statistical limitations that may hamper the statistical validity of the reported findings. Conclusion or statistical validity is the degree to which conclusions that we reach about relationships in our data are reasonable (Trochim, 2000). The first limitation concerns the response scale of the indicators in these measures. The response anchors often express a frequency of exposure like the following: ‘never,’ ‘occasionally,’ ‘often’ (‘monthly’), ‘weekly,’ and ‘always’ (or ‘daily’). Strictly speaking, such response anchors do not constitute an interval scale but should rather be treated as an ordinal scale (Hershcovis and Reich, 2013). A second limitation consists of measuring social stressors with anchor responses using a frequency count. This assumes that all incidents are equal in severity and interpretation (Notelaers et al., 2006a; Hershcovis and Reich, 2013), whereas it is reasonable to assume that different types of behaviors may have different consequences for those exposed (Hoel et al., 2004; Hershcovis and Reich, 2013).

To deal with the limitations mentioned above, and their resulting challenges, we propose the use of latent class cluster (LCC) and latent class factor (LCF) analysis. LC models are suitable for several reasons. Firstly, LC models can deal with count, continuous, interval, ordinal and nominal measures. Secondly, LC models can also take into account the fact that item properties, such as item difficulty and discriminatory power of items, may diverge (Vermunt, 2001). Thirdly, LC models do not depend strongly upon distributional assumptions (Magidson and Vermunt, 2002; Vermunt and Magidson, 2002) which is important in this field.

### Modeling a Latent Class Cluster or Factor Model

For the examination of the relationships between interpersonal conflicts, aggression and bullying items, the statistical software package Latent Gold 5.1 was used (Vermunt and Magidson, 2016). LC analysis is a useful statistical technique for clustering individuals into subtypes within a population when there is no prior knowledge about which individual belongs to which subpopulation. This method is used to analyze multi-variate categorical data and model associations between observed variables that provide an imperfect measure of a non-observable (latent) variable. LC analysis enables the researcher to identify mutually exclusive groups that adequately describe the dispersion of observations in the n-way contingency table of discrete variables (i.e., workplace bullying, aggression and conflict items). The goal of traditional LC analysis is to determine the smallest number of latent classes, sufficiently explaining (or accounting for) the associations observed between the manifest variables (all the items in our study) (Magidson and Vermunt, 2004). The traditional LC model (Goodman, 1974) assumes that every observation is an exclusive member of one latent (unobservable) class and that local independence exists between the manifest variables. LC analysis only assumes nominally distributed LC dimensions and binary or polytomous observations (Rist et al., 2009). An important difference from traditional cluster methods (like *K*-means clustering) is that LC analysis is based on a statistical model that can be tested (Magidson and Vermunt, 2002). As a consequence, determining the number of latent classes is less arbitrary than when using traditional cluster methods. In fact, LC analysis offers robust, empirically supported tests to determine the optimal number of classes.

The starting-point for a LC model is homogeneity, that is, every respondent resides in the same single group. This baseline model is a one-LCC model. In a LCC model, clusters of respondents with similar response patterns are subsequently added. A n-cluster model may then result in latent classes that differ in function of the nature and the frequency of reported social stressors. The metric of this single latent variable is typically nominal. This would fit the assumption that aggressive behavior manifests itself in different shapes and doses. Instead of increasing the number of LCCs only, the number of latent variables (factors) may be increased as well, addressing, in our case, the degree to which these three measures are representing either one or, rather, multiple factors. The idea of defining a LC model with several latent variables started with Goodman (1974), Haberman (1979), and Hagenaars (1990, 1993) who proposed restricted 4-class LC models yielding models with two latent variables. Magidson and Vermunt (2001) labeled this type of LC models as LCF models because of the natural analogy to standard factor analysis. Like with traditional confirmatory factor models, *a priori* knowledge about the relationship between items and latent variables is needed (Vermunt and Magidson, 2005). Moreover, with traditional measurement models, the (discrete) latent variable must adequately explain the initial relationship between the indicators. In an LC model, every subject is assigned to only one cluster or class based upon the modal assignment rule that classifies a subject to the class with the highest classification probability. These membership probabilities are being calculated upon the estimated parameters of the measurement model (Magidson and Vermunt, 2004).

Evaluation of fit of LC models is not straightforward. Firstly, the model fit needs to be evaluated. Secondly, the local fit has to be assessed and finally, the quality of the classification has to be scrutinized. For model selection, the Bayesian Information Criterion (BIC) is used. McCutcheon (1987) and Hagenaars (1990) suggest to select the model with the lowest BIC. After selecting a specific model, it is assessed whether it fits to the data. A model that does not fit to the data has a significant squared log-likelihood (L^2^). However, for very sparse tables such as the ones we have, Langeheine et al. (1996) suggested a bootstrapping procedure. In addition to statistical fit measures, it is also important to inspect local fit and the quality of the classification. To evaluate local fit or misfit and its origin one may use bivariate residuals (BVR). BVR show how much association between each pair of indicators remains, using the 1-cluster model as a reference. Ideally, the value should be lower than 3.84, being a value which corresponds to a significant χ^2^ with 1 degree of freedom (StaticalInnovations, 2013). However, as the L^2^ follows a χ^2^ distribution, the BVR is also quite sensitive for large sample size. Therefore, we suggest using a more relative threshold, where the reduction of the BVR should be at least 90% (Notelaers et al., 2006a). Finally, the quality of the classification is assessed. Here R^2^, entropy R^2^, and the total rate of classification errors, due to adjacent erroneous classifications, are indicators of (mis)classification.

Given the large sample size in our study, both BIC and L^2^ may lose their power to select the most appropriate model (Paas, 2014). The proper use of these statistical fit measures has only been illustrated for samples with a maximum of 500 respondents, leaving big data in the rain (Paas, 2014). Because the evaluation of fit and the comparison of fit between the different measurement models are central to evaluate our first research hypothesis, we randomly selected six mutually exclusive subsamples from the overall sample to investigate which of the models had the best fit to the data. Thereafter, we applied this model to the entire sample and studied the relationships between the constructs and their criterion validity.

## Results

### Identification of the Appropriate Measurement Model

To test the first hypothesis, we inspected the BIC across the six samples that were randomly selected (see first part of **Table 2**). The second part of **Table 2** portrays the BIC of the final model and the p-value of the bootstrapping procedure of the L^2^. To support Hypothesis 1, the BIC of the single latent variable model (LCC model) should be lower than the BIC of the competing factor models (Models 2–3). The BIC in **Table 2** shows that in only one of the six selected samples the BIC of the LCC model is lower than the BIC of the LCF models (Models 2–3). Hence, we reject Hypothesis 1 and conclude that the items measuring interpersonal conflicts, aggression and workplace bullying, respectively, do not fit into one single and unified concept.

**Table 2:** Evaluation of fit: Bayesian information criterion across different competing measurement models and samples.

Latent class model	S1	S2	S3	S4	S5	S6
Model 1 - 4cl	8936.9	9492.3	9227.7	8607.9	9483.5	9595.2
Model 1 - 5cl	8939.4	9498.0	9242.5	8624.0	9502.2	9624.5
Model 1 - 6cl	8960.4	9534.8	9267.8	8634.1	9531.7	9665.4
Model 1 - 7cl	8978.6	9580.3	9297.8	8664.2	9553.7	9449.8
Model 2a – CA - WB: (aggression and conflicts 4cl) - bullying 4cl	8831.1	9354.8	9138.2	8468.7	9386.2	9574.4
Model 2b AWB – C: conflicts 4cl - (aggression and bullying 4cl)	8902.6	9446.8	9214.6	8565.2	9425.1	9519.1
Model 2c A – CWB: aggression 4cl - (conflicts and bullying 4cl)	8916.9	9463.9	9229.9	8565.6	9476.8	9551.1
Model 3 A – C – WB. conflicts 4cl - aggression 4cl - bullying 4cl	8865.4	9388.0	9173.0	8503.7	9405.7	9468.2
**Final model**						
(aggression and conflicts 3cl) - bullying 4cl	8848.1	9361.14	9145.984	8495.9	9398.1	9487.037
Bootstrap (L^2^) *p*-value	0.1	0.098	0	0.158	0.138	0.112


*S, Sample; BIC(LL), Bayesian Information Criterion based on the Log-Likelihood.*

In addition, the BIC showed that any of the distinguished multi-dimensional factor models had a lower BIC than the single variable latent model. The most plausible model among these alternative measurement models was a two-LCF model (Model 2a– CA-WB) wherein bullying represents one factor with different classes, and wherein conflicts and aggression represent a second factor again with different latent classes. This model portrayed the lowest BIC in 5 out of 6 subsamples. According to the BIC statistics, the second best model distinguished between all three factors (Model 3– A-C-WB).

The two-factor model with the lowest BIC across the six samples distinguished four latent classes for each factor. As one of the classes in the conflicts-aggression factor was relatively small (3.6% on average), consisting of respondents who hardly reported any exposure to the indicated problems and therefore had little added meaning over and above the other classes, we decided to estimate a model that only differentiated between three classes for the conflicts/aggression cluster. Apart from the previous model, this final model had the lowest BIC and its bootstrapped L^2^ was also non-significant in 5 out of 6 samples, indicating that this measurement model statistically fits well to the data.

**FIGURE 1:**
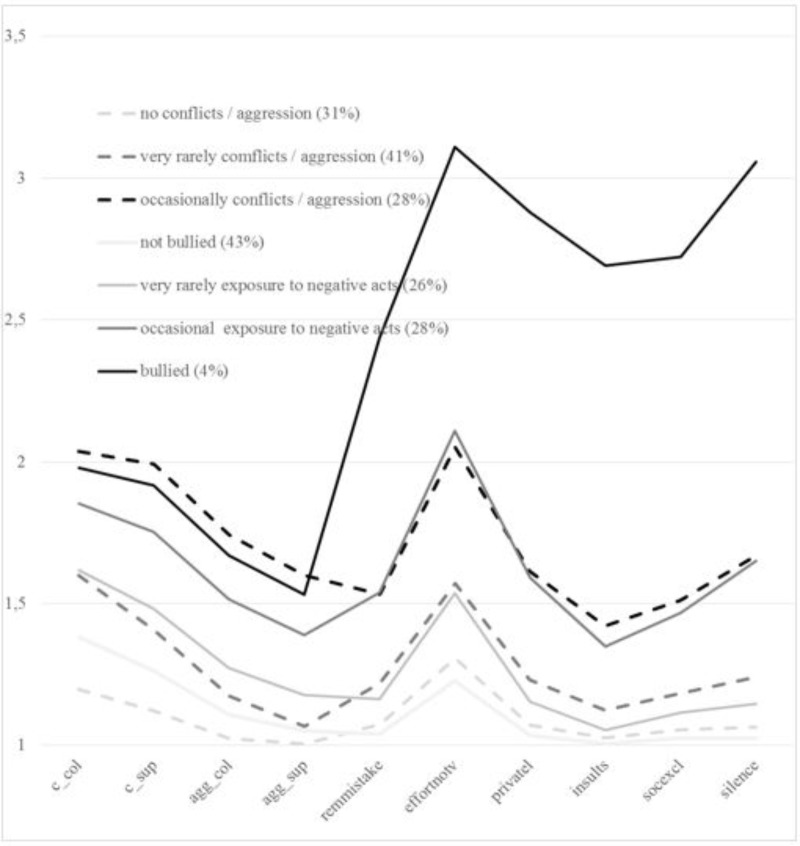
Conditional means plot. X-axis: c_col: conflict with colleagues; c_sug: conflict with supervisor; agg_col: aggression from colleagues; agg_sup: aggression from supervisor; remmistake: repeated reminders about your mistakes; effortnov: your effort is not valued; privatel; remarks about your private life; insults: insults; socexcl: social exclusion; silence: hostility of silence at you attempts to start a conversation or when you approach. Y-axis 1: never 2: occasionally 3: often / monthly 4: always / weekly or more often.

The precise meaning of the latent classes of the two factors can be derived from the conditional probabilities. However, for the sake of economic expression (the full table has 8 columns and 41 rows covering 280 conditional probabilities and is portrayed in the **Annex**) we limit us to **Figure 1** where we have plotted the conditional average scores of the items across the LCFs. The dashed lines represent the classes of the conflicts-aggression factor whereas the full lines represent the classes of the bullying factor. The first four items on the left-hand side of **Figure 1** are the indicators for the conflicts/aggression factor, while the other six items are the indicators for the bullying factor. The percentages in **Figure 1** correspond to the size of the latent classes.

The conflicts/aggression factor consisted of three latent classes. In the first class, neither conflicts nor exposure to aggression were reported. We therefore labeled this class of respondents with *“never conflicts nor aggression.”* In the second class, conflicts and aggression were reported slightly more often, although the frequency remained very low. We decided to label this class *“rarely conflicts.”* In the third class, the average of the conflicts and aggression items was somewhat higher. More specifically, whereas both conflict items were occurring on average ‘sometimes,’ both aggression items were occurring slightly less frequently. We suggest labeling this class as *“occasionally conflicts and aggression.”*

The bullying factor consisted of four latent classes. In the first class, hardly any exposure to bullying behaviors was reported, leaving us with the label of *“never bullied”* for the respondents in this class. In the second class, there was a slightly higher, yet still low, frequency of reported exposure to the bullying items. We decided to label this class *“hardly any bullying behaviors.”* In the third class, the average of the six items was considerable higher and even close to 2. Therefore, we suggest labeling this class *“occasionally bullied.”* Finally, in the fourth class, all averages were well above 2, indicating that the frequency of bullying behavior was highest here. Thus, we suggest labeling this class *“targets of bullying.”*

**FIGURE 2:**
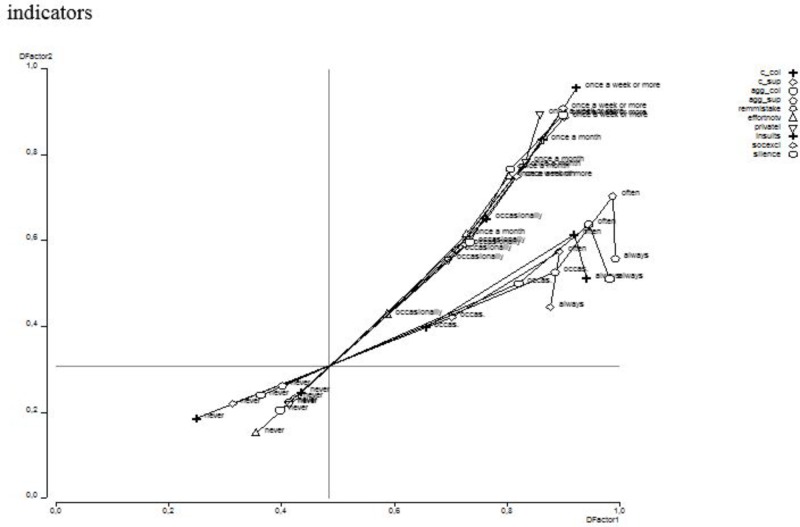
Bi-plot: relationship between conflict-aggression and workplace bullying and their indicators. DFactorl: conflict-aggression; DFactor2: Bullying at work; Numbers on the X and Y portray probability to score high on the DFactor. c_col: conflict with colleagues; c_sup: conflict with supervisor; agg_col: aggression from colleagues; agg_sup; aggression from supervisor; remmistake; repeated reminders about your mistakes; effortnov: your effort is not valued; privatel: remarks about your private life; insults: insults; socexcl: social exclusion; silence: hostility of silence at you attempts to start a conversation or when you approach.

The two factors (i.e., conflicts/aggression and bullying) were significantly related to each other with their ordinal correlation being 0.644. **Figure 2** sheds more light on the relationship between both factors via a bi-plot. The bi-plot is analogous to a factor plot because it depicts the relationship between the responses to the indicators of the conflicts/aggression factor, on the one hand, and the relationship between the responses to the indicators of the bullying factor, on the other hand, as well as the relationship between both factors. The bi-plot indicates that the less frequently conflicts/aggression and bullying were reported, the stronger the relationship was between both factors. From studying the response categories of conflict and aggression on an item-level it becomes obvious that when conflicts and aggression were ‘always’ present, it is less likely that these coincide with reported exposure to bullying. This tendency also seems to exist for bullying at work. This implies that the strength of the relationship between the two factors decreased as conflict, aggression, and bullying were reported more frequently.

The findings presented in **Table 3** allow us to further elaborate on the relationship between the two factors. The cross-tabulation in **Table 3** again demonstrates that the overlap between the two constructs is large (over 90%) when the problems are reported to happen rather infrequently. However, when both behaviors become more frequent (occasional and more often), their overlap decreased. Furthermore, it seems that practically all targets of bullying also experienced occasional conflicts/aggression, while, ‘only’ one out of eleven of the employees experiencing the latter was a target of bullying.

**Table 3:** Estimate values model: relationship between both latent class factors.

	Not bullied	Very rarely NB	Occasionally bullied	Target of bullying	Total
Nor aggression nor conflicts	24.76	5.3	0.9	0.01	30.95
Rarely conflicts	16.38	14.53	9.98	0.46	41.35
Occasional conflicts –aggression	1.62	5.95	16.94	3.21	27.72
Total	42.76	25.78	27.8	3.68	100.00


### Criterion Validity

To test the second hypothesis stating that conflicts, aggression, and bullying are similarly detrimental to those exposed, we disentangled the effect size of both factors in a multi-variate analysis of variance. The partial eta^2^ resulting from the multi-variate analysis of variance helps to understand the predictive value of the two factors in explaining the various criterion variables. If the partial eta^2^ for the bullying factor is higher than the one for the conflicts/aggression factor, we can argue that we should reject Hypothesis 2.

**Table 4** shows the magnitude of the relationships between the two factors (conflicts/aggression and bullying), on the one hand, and with the outcome variables, on the other hand. Results show that the relationships with the outcome variables are stronger for the bullying factor than for the conflicts/aggression factor, except for “worrying.” Hence, given these outcomes, we have to reject Hypothesis 2 as well. After assessing the effect sizes of both factors, we further discerned the mean differences of the latent classes of both factors on the criterion variables. A Tukey pair-wise comparison procedure yielded that all pair-wise comparisons were significantly different from each other for all criterion variables. **Table 5** enlists the specific *z*-scores of the criterion variables, for each class separately. For the sake of simplicity, we did not print the “not exposed” classes. **Table 5** demonstrates that the effects of reporting conflicts/aggression occasionally and reporting exposure to negative acts occasionally are similar. However, being classified as a target of bullying seems to be responsible for a further deterioration of job satisfaction etcetera as the z-values increase (on average, with 0.5 standard deviations). Hence, targets of bullying report significantly more detrimental outcomes than respondents who are most strongly exposed to interpersonal conflicts and aggression.

**Table 4:** Partial eta squared.

	Conflicts-aggression	Bullying
Job satisfaction	0.001	0.041
Affective commitment	0.004	0.018
Turnover intention	0.004	0.020
Recovery need	0.005	0.030
Worrying	0.011	0.012
Sleep quality	0.002	0.037


**Table 5:** Tukey pair wise comparison.

Criterion	Bullying classes	*z*-values	Conflict and aggression classes	*z*-values
Job satisfaction	Rarely negative behaviors	0.007	Rarely conflict	–0.220
	Occasionally bullied	–0.290	Occasional conflict rarely aggression	–0.302
	Target	–0.870		
Organizational commitment	Rarely negative behaviors	0.004	Rarely conflict	–0.171
	Occasionally negative behaviors	–0.188	Occasional conflict rarely aggression	–0.233
	Target	–0.537		
Turnover	Rarely negative behaviors	–0.038	Rarely conflict	–0.251
	Occasionally negative behaviors	–0.151	Occasional conflict rarely aggression	–0.270
	Target	–0.733		
Recovery need	Rarely negative behaviors	–0.012	Rarely conflict	–0.169
	Occasionally negative behaviors	–0.219	Occasional conflict rarely aggression	–0.317
	Target	–0.705		
Worrying	Rarely negative behaviors	0.019	Rarely conflict	–0.145
	Occasionally negative behaviors	–0.126	Occasional conflict rarely aggression	–0.336
	Target	–0.550		
Sleep quality	Rarely negative behaviors	–0.004	Rarely conflict	–0.202
	Occasionally negative behaviors	–0.259	Occasional conflict rarely aggression	–0.308
	Target	–0.816		


*All mean differences are significantly different.*

## Discussion

Construct proliferation is a major problem in the organizational sciences, and research on conflict, aggression, and bullying might not be immune to that problem (Aquino and Thau, 2009; Hershcovis, 2011; Tepper and Henle, 2011; Hershcovis and Reich, 2013). However, existing research may not be fine-grained enough to distinguish clearly between the different concepts (Tepper and Henle, 2011). Here, we provide one of the very first empirical tests of the convergence/divergence of workplace conflict, aggression, and bullying. We examined a set of competing LC models using a large heterogeneous sample of Belgian workers. While comparing LC models, we found our data to not support an approach where labels could be used interchangeably (i.e., unifying approach). From a statistical point of view, a two-factor model fitted the data best—with one factor comprising both conflict and aggression and another one comprising bullying. A three-factor model only provided the second-best solution. Furthermore, we found that the effect sizes for the bullying factor regarding selected criterion variables were larger than the effect sizes for the conflict/aggression factor. Notably, even when the effect sizes of the conflict/aggression factor were significant, they were close to zero. Hence, it appears that bullying is not only perceived differently than aggression and conflict but also seems to have a unique impact on employees, which seems to be especially detrimental for those employees that are highly targeted. Although more difficult to differentiate between the three types of social stressors for lower intensity levels, we found that when reported more frequently, interpersonal conflict, aggression and bullying cannot easily be construed as the same underlying phenomenon. Employees who are targets of bullying experience this as something quite different than being ‘occasionally’ in conflicts or experiencing ‘merely’ aggression at work. Moreover, the targets of severe bullying reported by far the lowest levels of well-being and the highest levels of strain among all identified classes of respondents. Hence, being a target of severe bullying seems to constitute a discrete experience associated with particularly low levels of well-being and particularly elevated levels of work-related strain.

Introducing a separate factor for aggression did not improve model fit. Aggression was primarily reported in situations where interpersonal conflicts exist between subordinates and superiors or between peers. This might imply that interpersonal conflicts might encompass workplace aggression. Said differently, “workplace aggression may be construed as a particular form of conflict” (Raver and Barling, 2008, p. 222), or at least as something perceived to take place within the context of interpersonal work conflicts. Note that we do not claim that all conflicts involve aggression, as we also found some instances where conflicts did exist without any trace of aggression, particularly at low levels of conflict.

Even though we found a two-factor solution providing the best fit to the data, the overlap between these two factors appeared to be quite large, with the correlation between the two being almost 0.65. The scores on the selected criterion variables for those experiencing some involvement in interpersonal conflicts or for those who were occasionally bullied were also quite similar. Hence, it seems that both phenomena are not that different when exposure is low. The cross-tabulation table (see **Table 2**) further demonstrates that not all conflicts involve bullying, but that targets of bullying tend to experience some degree of interpersonal conflict. This outcome is not surprising in light of Thomas (1992) who defined conflict as “the process that begins with a party perceiving that the other party has negatively affected or is about to negatively affect, something he or she cares about” (p. 653).

Existing research may help explain the interrelations between conflict and bullying. Einarsen (2005) proposed a model wherein these two processes are strongly interrelated. His model starts with an escalating conflict which may provoke aggression, which, in turn, may result in bullying. The idea that conflicts may escalate to a level where aggressive behaviors come to the fore is in alignment with the latent classes of the conflict/aggression factor we found. The first class was typified by not reporting any conflicts and no aggressive behavior. The second class was typified by increasing level of conflicts but hardly any aggressive behavior. Compared to the previous class, the last class consisted of employees reporting increasing levels of conflicts and also aggressive behaviors. That aggression may coalesce with elevated levels of conflicts fits with Glasl’s (1994) conflict escalation model wherein parties evolve from a win-win to a win-lose situation. Workplace attitude and well-being was substantially lower in the “conflict/aggression” classes as compared to the “neither conflict nor aggression” class.

Nevertheless, the class that is missing in the current results is the one that describes a stage of conflict escalation wherein the conflicting parties go so far that they envisage total annihilation. According to Einarsen and colleagues (2011), it is unlikely to find such highly escalated interpersonal conflicts while at work. A reason for why we did not find this class may be that such highly escalated conflicts are most likely to be stopped by management. Furthermore, in general, such intense overt aggressive behaviors will not be tolerated in working life. Such types of behaviors, as they are illegal, may even warrant dismissal (Welzijnswet, 1996), which explains why this class may be rare in working life. However, to the extent that parties engage in subtle, covert, and difficult-to-detect wrong-doing, such behavior may persist for long, as often the case with bullying. For example, dispute-related bullying describes a form of bullying that develops out of grievances and involves social control reactions to perceived wrong-doing (Einarsen, 1999; also see Felson and Tedeschi, 1993). Einarsen et al. (1994) and Leymann (1996) claim that bullying may be triggered by a work-related conflict, and that in some instances the social climate at work turns out to be more than just sour, which, as a result, creates conflicts that may escalate into harsh personified conflicts (van de Vliert, 1984). At such an escalated level, parties may subsequently deny their opponent’s human value, herewith clearing the way for manipulation, retaliation, elimination and destruction (van de Vliert, 1984). If one of the parties involved already has or will acquire a disadvantaged position, he or she may become a victim of workplace bullying (Björkqvist et al., 1994a,b). Hence, a certain critical level of interpersonal conflicts may give rise to a process of escalating bullying.

If dispute-related bullying would be the only way for bullying to emerge, our empirically tested cluster model wherein conflicts, aggression and bullying are indicators of the same phenomenon should have had the best fit. That this is not the case is potentially due to the existence of predatory bullying, which describes a form of workplace bullying that gradually evolves in the absence of (an escalated) conflict. Previously, scholars already distinguished between different phases in this process. During the early phases of bullying, targets are typically subjected to negative social behaviors that are difficult to pinpoint as the perpetrator is still very indirect and discrete (Björkqvist, 1992). Later on, more direct negative social behaviors seem to appear (Einarsen et al., 2011). Targets are isolated and avoided, humiliated in public by being made a laughing-stock, and so on. In this phase both physical and psychological means of violence may be used (Einarsen et al., 2011). Our results may coincide with earlier empirical research using a LC approach that seems to underwrite such a description of the process of bullying – note though that such a process cannot be modeled with cross-sectional data. The increases in conditional probabilities between the different levels of bullying classes do however, point to such a process. In the occasionally bullied class, the conditional probabilities to report having more often experienced repeated reminders about mistakes, and remarks about one’s private life increased compared to the previous classes. Finally, similar to other studies, the target of bullying class yielded the highest conditional probability to report the most frequent exposure to all types of negative behaviors.

### Strengths and Limitations

Our re-analysis of the extensive data used originally by Notelaers et al., 2006a,b goes beyond existing work in that we simultaneously account for conflict, aggression, and bullying, and their workplace effects. Although clearly advancing prior research, our study is not without limitations. Our data is constraint by the fact that conflict and aggression were only measured with two items each. Although the global and the local fit were sufficient, measuring latent variables with only two indicators is sub-optimal because it is generally known that it takes three indicators to identify a factor using covariance modeling. Another possible limitation is that the number of items to measure each construct may be imbalanced. Specifically, one may question whether the established two-factor solution may be a consequence of the fact that we used more items to measure bullying than conflict and aggression. Researchers should replicate our findings by using more established and more extensive conflict and aggression measures. Finally, it may be that respondents are more familiar with conflict and aggression than with bullying. Although research has shown that such a bias did not play in the Scandinavian countries (Nielsen, 2009), we cannot rule out that this may be a factor that influences the prevalence in other countries.

Due to the cross-sectional nature of our data, we cannot investigate possible escalation processes, which theoretically, are assumed to be central to the understanding of both escalated conflicts and to workplace bullying. The lack of longitudinal data also defers conclusions on the cause and effect relationships between the social stressors and the included outcome variables.

Furthermore, we were unable to assess the validity of the identified classes by means of any objective measure. Future research may use existing registries of interpersonal conflicts and aggression incidents, complaints about workplace bullying, and third party measures (León-Pérez et al., 2016). This would also help to develop a more profound understanding of the nomological network of social stressors at work. With respect to the nomological network, we urge scholars to not only use alternative measures and methods to measure conflicts, aggression and bullying but also to further investigate the convergent and divergent validity of these three concepts.

### Future Directions

In this study, we focused on the target of conflict, aggression, and bullying, but not the offender (and/or bystanders). While the target perspective is the dominant one in risk management research on interpersonal conflicts and workplace bullying, it limits research as conflict, aggression, and bullying involve at least two parties, and sometimes also bystanders (Branch et al., 2009). An interesting but under investigated question is whether offenders and bystanders construe negative social workplace behaviors in a similar way as targets do. Recent research findings indeed have shown that targets and witnesses react differently toward the display of negative social workplace behavior (Nielsen and Einarsen, 2013).

Future research also is needed to clarify the nomological network of conflict, aggression, and bullying (Hershcovis, 2011; Tepper and Henle, 2011). The current study together with that of Baillien et al. (2017)—studying the experience involved in interpersonal conflicts and bullying—provide an important step in this direction. To further establish construct validity and ascertain that one is not using different labels for the same construct, we need, however, more than valid statistical methods and existing data. Campbell and Fiske (1959) bring the importance of discriminant and convergent validity to our attention when presenting the multi-trait multi-method matrix. Recently Baillien et al. (2017), found targets of bullying, as compared to employees involved in conflicts, to report more frequent conflicts, a higher exposure to negative social behaviors, and stronger power imbalance. They also perceived bullying to be a longer-lasting process that was governed by an intent to harm. Future research aiming to advance research on the construct validity of the three concepts could operationalize these characteristics together with the characteristics that are typical for aggression and conflicts. This would allow an in-depth study of the convergent and discriminant validity.

### Practical Implications

Our study findings provide some guidance for managers and policy makers who seek to prevent and manage workplace conflict, aggression, and bullying. Our findings suggest that companies, at a minimum, would need to raise awareness for the potentially grave implications of conflict, aggression, and bullying and would need to design policies to help prevent those social stressors at work from occurring in the first place. This involves that employees may need to learn about conflict de-escalation strategies (De Dreu and Gelfand, 2007) and receive conflict management trainings that help prevent conflicts from escalating (León-Pérez et al., 2016). Employees who are occasionally bullied should receive individual counseling to help them cope with the situation. In cases of severe bullying, however, counseling will not suffice. Thus, companies need to develop and enforce legal procedures to help protect targets of bullying (Hoel and Einarsen, 2010; Yamada, 2010). Managers also need to be aware that they may do more harm than good when confusing incidents of conflicts (or aggression) with examples of workplace bullying, and vice versa. A strategy merely aimed at solving a “conflict” would not be appropriate if the conflict actually is an example of bullying, and such “conflict management interventions” may be resented by those employees being bullied. The challenge for managers thus is to learn to tell apart workplace conflicts from bullying incidents, as both kinds of social stressors afford different kinds of interventions (Hoel and Einarsen, 2011).

## Conclusion

In this article, we have addressed a rather straightforward research question that is relevant for both theorists and practitioners dealing with social stressors at work: Are interpersonal conflicts, aggression and bullying at work different or overlapping phenomena, at least as experienced and perceived by those exposed, and, are their outcomes similar or different? Our findings from a LC analysis speak against using these labels interchangeably. While interpersonal conflicts and aggression are strongly intertwined, workplace bullying is construed as a distinct concept. The results of the present study show that severe forms of workplace bullying are not seen by the targets as being merely another kind of interpersonal conflicts at work or as a case of aggression, but that they rather constitute a distinct phenomenon associated with even more severe outcomes. Furthermore, the large majority of employees who experience conflicts did not report any exposure to bullying. Our findings have important implications for practitioners and researchers. We call for more attention for this topic in order to prevent unnecessary human suffering at work and to enable sustainable employability (van der Heijden, 2015).

## Ethics Statement

No review and approval from an ethical board was required according to the relevant institutional and national guidelines. For a study that does not comprise of any health related an informed consent was neither needed according to the institutional and national standards of Belgium in 2006. However, respondents were informed about the goal of the project, that they were added to the benchmark and the use of the latter for scientific research. They had after completing the questionnaire that in total had 256 items over 14 pages, to either put it sealed envelope and to put in a sealed urn or sent it back to the Health and Safety Executive or to the Directorate Research of Working Conditions of the Belgian Federal Department of Labor. This Federal Research Institute treated the data and made reports for the HSEs in order to ensure their further commitment to future studies on psychosocial factors. Participation of the specific individual employees was voluntary and anonymity was guaranteed by all parties involved. None of the members of the participating organizations had access to any of the completed surveys, herewith fully guaranteeing anonymity. Person data was made anonymous which is also in line with the current European General Data Protection Act.

## Author Contributions

GN, MN, and SE designed the study. GN has collected and analyzed the data. GN, MN, SE, HG, and BVdH edited the manuscript. GN, SE, HG, and BVdH reedited the manuscript.

## Conflict of Interest Statement

The authors declare that the research was conducted in the absence of any commercial or financial relationships that could be construed as a potential conflict of interest.
