# Supplementary material for: Do Interpersonal Conflict, Aggression and Bullying at the Workplace Overlap? A Latent Class Modeling Approach
Source: Front Psychol. 2018 Oct 9;9:1743. doi: 10.3389/fpsyg.2018.01743 (PMC6189319; doi:10.3389/fpsyg.2018.01743)
Supplement: Supplementary file 1 [file Data_Sheet_1.DOCX]

**Annex**

Table 1. Conditional probabilities

|  | Conflict-Aggression | | | Bullying | | | |
| --- | --- | --- | --- | --- | --- | --- | --- |
|  | never aggression nor  conflicts | rarely conflicts | ocassional conflicts and aggression | not bullied | rarely exposure to negative behaviours | occasionally bullied | target  of bullying |
| Class Size | 0.3094 | 0.4135 | 0.2771 | 0.4276 | 0.2578 | 0.2779 | 0.0367 |
| Indicators |  |  |  |  |  |  |  |
| c_col |  |  |  |  |  |  |  |
| never | 0.8027 | 0.4091 | 0.0974 | 0.6252 | 0.418 | 0.2316 | 0.138 |
| occasionally | 0.1968 | 0.5826 | 0.8059 | 0.3677 | 0.5549 | 0.7065 | 0.7766 |
| often | 0.0004 | 0.0075 | 0.0601 | 0.0054 | 0.0182 | 0.0393 | 0.0534 |
| always | 0 | 0.0008 | 0.0366 | 0.0017 | 0.0089 | 0.0226 | 0.032 |
| Mean | 0.1977 | 0.6 | 1.0358 | 0.3835 | 0.6179 | 0.8529 | 0.9796 |
| c_sup |  |  |  |  |  |  |  |
| never | 0.8797 | 0.6089 | 0.2214 | 0.751 | 0.5751 | 0.3813 | 0.2713 |
| occasionally | 0.1194 | 0.3765 | 0.6237 | 0.237 | 0.3807 | 0.5191 | 0.5917 |
| often | 0.0009 | 0.0129 | 0.0973 | 0.0091 | 0.0299 | 0.064 | 0.0866 |
| always | 0 | 0.0017 | 0.0575 | 0.0028 | 0.0142 | 0.0356 | 0.0504 |
| Mean | 0.1213 | 0.4073 | 0.9909 | 0.2638 | 0.4832 | 0.7539 | 0.9162 |
| agg_col |  |  |  |  |  |  |  |
| never | 0.9743 | 0.8288 | 0.3582 | 0.8953 | 0.7501 | 0.5466 | 0.4182 |
| occasionally | 0.0256 | 0.1691 | 0.5664 | 0.101 | 0.2313 | 0.4067 | 0.5156 |
| often | 0 | 0.002 | 0.0515 | 0.0027 | 0.013 | 0.0321 | 0.0452 |
| always | 0 | 0.0001 | 0.024 | 0.001 | 0.0056 | 0.0146 | 0.0209 |
| Mean | 0.0257 | 0.1734 | 0.7411 | 0.1094 | 0.2741 | 0.5147 | 0.6688 |
| agg_sup |  |  |  |  |  |  |  |
| never | 0.9933 | 0.9327 | 0.5206 | 0.9522 | 0.85 | 0.6835 | 0.573 |
| occasionally | 0.0067 | 0.0662 | 0.3888 | 0.044 | 0.1284 | 0.2609 | 0.3477 |
| often | 0 | 0.001 | 0.061 | 0.0027 | 0.0146 | 0.0375 | 0.0534 |
| always | 0 | 0 | 0.0296 | 0.0011 | 0.0069 | 0.0181 | 0.0259 |
| Mean | 0.0067 | 0.0683 | 0.5997 | 0.0528 | 0.1783 | 0.3902 | 0.5322 |
| Remmistake | |  |  |  |  |  |  |
| Never | 0.9268 | 0.8014 | 0.5661 | 0.9603 | 0.8398 | 0.5154 | 0.1273 |
| occasionally | 0.071 | 0.1819 | 0.3604 | 0.0395 | 0.157 | 0.4377 | 0.4912 |
| once a month | 0.0018 | 0.0128 | 0.0476 | 0.0002 | 0.0031 | 0.0391 | 0.1992 |
| once a week or more | 0.0003 | 0.004 | 0.0259 | 0 | 0.0001 | 0.0079 | 0.1823 |
| Mean | 1.0756 | 1.2193 | 1.5334 | 1.0399 | 1.1636 | 1.5394 | 2.4365 |
| Effortnotv |  |  |  |  |  |  |  |
| Never | 0.7144 | 0.5365 | 0.2838 | 0.7766 | 0.5093 | 0.205 | 0.0331 |
| occasionally | 0.268 | 0.3861 | 0.4929 | 0.2181 | 0.4509 | 0.5722 | 0.2908 |
| once a month | 0.0133 | 0.0471 | 0.111 | 0.005 | 0.0325 | 0.1302 | 0.2086 |
| once a week or more | 0.0043 | 0.0302 | 0.1123 | 0.0004 | 0.0073 | 0.0925 | 0.4675 |
| Mean | 1.3076 | 1.571 | 2.0517 | 1.2291 | 1.5379 | 2.1102 | 3.1105 |
| Privatel |  |  |  |  |  |  |  |
| Never | 0.9324 | 0.8053 | 0.5602 | 0.9652 | 0.8508 | 0.5095 | 0.0846 |
| occasionally | 0.0641 | 0.1668 | 0.3206 | 0.0346 | 0.1444 | 0.4096 | 0.3219 |
| once a month | 0.0027 | 0.0186 | 0.0633 | 0.0002 | 0.0045 | 0.0599 | 0.2228 |
| once a week or more | 0.0008 | 0.0093 | 0.0558 | 0 | 0.0003 | 0.021 | 0.3707 |
| Mean | 1.0719 | 1.2318 | 1.6148 | 1.0351 | 1.1543 | 1.5924 | 2.8797 |
| Insults |  |  |  |  |  |  |  |
| Never | 0.9751 | 0.8919 | 0.6933 | 0.9922 | 0.9452 | 0.6851 | 0.1188 |
| occasionally | 0.0237 | 0.0949 | 0.228 | 0.0078 | 0.0542 | 0.2852 | 0.3594 |
| once a month | 0.0009 | 0.0089 | 0.0426 | 0 | 0.0007 | 0.0254 | 0.2326 |
| once a week or more | 0.0002 | 0.0042 | 0.0361 | 0 | 0 | 0.0043 | 0.2892 |
| Mean | 1.0262 | 1.1255 | 1.4215 | 1.0079 | 1.0555 | 1.349 | 2.6922 |
| Socexcl |  |  |  |  |  |  |  |
| Never | 0.9476 | 0.8433 | 0.6289 | 0.9734 | 0.886 | 0.5998 | 0.1321 |
| occasionally | 0.0502 | 0.1375 | 0.2792 | 0.0264 | 0.1112 | 0.3481 | 0.3544 |
| once a month | 0.0016 | 0.0116 | 0.0427 | 0.0001 | 0.0025 | 0.0365 | 0.1717 |
| once a week or more | 0.0006 | 0.0076 | 0.0492 | 0 | 0.0002 | 0.0157 | 0.3418 |
| Mean | 1.0552 | 1.1836 | 1.5122 | 1.0267 | 1.117 | 1.4681 | 2.7233 |
| Silence |  |  |  |  |  |  |  |
| Never | 0.9392 | 0.7941 | 0.5156 | 0.9751 | 0.855 | 0.4424 | 0.0409 |
| occasionally | 0.0579 | 0.1789 | 0.3591 | 0.0248 | 0.1421 | 0.4803 | 0.2903 |
| once a month | 0.0023 | 0.0183 | 0.0654 | 0.0001 | 0.0027 | 0.0607 | 0.2397 |
| once a week or more | 0.0006 | 0.0088 | 0.0598 | 0 | 0.0001 | 0.0166 | 0.429 |
| Mean | 1.0644 | 1.2418 | 1.6695 | 1.025 | 1.148 | 1.6516 | 3.0568 |

Legend :

c_col: conflict with colleagues; c_sup: conflict with supervisor; agg_col: aggression from colleagues; agg_sup: aggression from supervisor; remmistake: repeated reminders about your mistakes; effortnov: your effort is not valued; privatel: remarks about your private life; insults: insults; socexcl: social exclusion; silence: hostility of silence at you attempts to start a conversation or when you approach.

Note : the conditional probabilities for each cluster or class  portray the relationship between the item responses and the measurement model. For instance  for each bullying class the table contains the conditional probability (CP) of responding ‘Never’, ‘Now and then’, ‘Once a month’ or ‘Once a week or more’ for each item. The means are portrayed in figure 1.
